# Mural granulosa cell gene expression associated with oocyte developmental competence

**DOI:** 10.1186/1757-2215-3-6

**Published:** 2010-03-06

**Authors:** Jin-Yi Jiang, Huiling Xiong, Mingju Cao, Xuhua Xia, Marc-Andre Sirard, Benjamin K Tsang

**Affiliations:** 1 Department of Obstetrics & Gynecology and Cellular & Molecular Medicine, University of Ottawa, Ottawa Hospital Research Institute, Ottawa, ON K1Y 4E9, Canada; 2 Department of Biology and Center for Advanced Research in Environmental Genomics, University of Ottawa, Ottawa, ON K1N 6N5, Canada; 3 Centre de Recherche en Biologie de la Reproduction, Département de Sciences Animales, Université Laval, Ste-Foy, QuébecG1K 7P4, Canada; 4 WCU Biomodulation Major, Department of Agricultural Biotechnology, Seoul National University, Seoul 151-921, Korea

## Abstract

**Background:**

Ovarian follicle development is a complex process. Paracrine interactions between somatic and germ cells are critical for normal follicular development and oocyte maturation. Studies have suggested that the health and function of the granulosa and cumulus cells may be reflective of the health status of the enclosed oocyte. The objective of the present study is to assess, using an *in vivo *immature rat model, gene expression profile in granulosa cells, which may be linked to the developmental competence of the oocyte. We hypothesized that expression of specific genes in granulosa cells may be correlated with the developmental competence of the oocyte.

**Methods:**

Immature rats were injected with eCG and 24 h thereafter with anti-eCG antibody to induce follicular atresia or with pre-immune serum to stimulate follicle development. A high percentage (30-50%, normal developmental competence, NDC) of oocytes from eCG/pre-immune serum group developed to term after embryo transfer compared to those from eCG/anti-eCG (0%, poor developmental competence, PDC). Gene expression profiles of mural granulosa cells from the above oocyte-collected follicles were assessed by Affymetrix rat whole genome array.

**Results:**

The result showed that twelve genes were up-regulated, while one gene was down-regulated more than 1.5 folds in the NDC group compared with those in the PDC group. Gene ontology classification showed that the up-regulated genes included lysyl oxidase (*Lox*) and nerve growth factor receptor associated protein 1 (*Ngfrap1*), which are important in the regulation of protein-lysine 6-oxidase activity, and in apoptosis induction, respectively. The down-regulated genes included glycoprotein-4-beta galactosyltransferase 2 (*Ggbt2*), which is involved in the regulation of extracellular matrix organization and biogenesis.

**Conclusions:**

The data in the present study demonstrate a close association between specific gene expression in mural granulosa cells and the developmental competence of oocytes. This finding suggests that the most differentially expressed gene, lysyl oxidase, may be a candidate biomarker of oocyte health and useful for the selection of good quality oocytes for assisted reproduction.

## Introduction

Ovarian follicle development is a complex process. Paracrine interactions between somatic and germ cells are critical for normal follicular development [1]. Defects in meiotic maturation have been observed in mice lacking the granulosa cell oocyte junction protein connexin 37 [2], and somatic cells in ovaries are known to participate in regulating oocyte growth and development [3,4], meiosis [5], and global transcriptional activity [6,7]. On the other hand, oocytes also promote granulosa cell proliferation and differentiation [1]. It has been shown that mouse oocytes promote granulosa cell proliferation in preantral and antral follicles in vitro [8] and that cumulus expansion and granulosa cell differentiation are dependent upon oocyte-derived factors [9,10]. In rodents, oocyte-secreted GDF-9 and BMP15 promote proliferation of granulosa cells from small antral follicles, and BMP15 inhibits FSH-stimulated progesterone production [11]. Evidence also indicates that while GDF9 suppresses expression of both *KitL-1 *and *KitL-2 *in granulosa cells from rat early antral follicles, KitL-1 expression can be promoted by BMP15 *in vitro *[4]. In addition, we have recently shown that GDF-9 from the oocyte promotes pre-antral follicles development by up-regulating granulosa cell FSH receptor mRNA expression and preventing granulosa cell apoptosis via activation of the phosphatidylinositol 3-kinase/Akt pathway [12]. Thus, while oocyte maturation is known to depend on secretory products of the granulosa and cumulus cells, proliferation, differentiation and apoptosis of these support cells is also under tight control of the oocyte, suggesting that the health and function of the granulosa and cumulus cells may be reflective of the health status of the enclosed oocyte.

The quality of the oocyte is largely dependent on its follicular environment, as shown in a number of animal and human studies [4,13]. During ovarian stimulation and ovulation induction, a cohort of heterogeneous follicles is recruited to develop and ovulate, irrespective of their differentiative state. This creates an asynchrony in the maturation process and heterogeneity in the quality of the oocytes recovered for assisted reproduction. The morphological appearance, which is widely used as the primary criterion for oocyte selection in the human fertility clinic, does not accurately predict the health of the oocyte [14]. In fact, only a small proportion of the oocyte population can develop to healthy embryos after fertilization and healthy fetuses after transfer.

Although multiple factors are at play in determining pregnancy outcome in assisted reproduction including age, sperm quality (male factor), fertilization capacity and number of embryos transferred, the effect of fertilization rate appears to be of less significance [15] and that intrinsic deficiencies of the oocyte and/or embryo account for greater than 50% of failed conceptions [16]. These findings suggest that the developmental competence of the oocytes is a major determinant in the establishment of successful pregnancy in assisted reproduction.

Two factors contributing to oocyte health are chromosomal constitution and gene expression patterns of the oocyte and the follicular micro-environment in which the oocyte grows and matures. It has been shown that eCG stimulates follicular development and oocyte maturation in immature rats [17]. After hCG treatment, superovulated oocytes in eCG-primed immature rats can be fertilized in vitro and developed to term after embryo transfer [18]. In addition, our model also indicates that eCG/hCG treatment resulted in decreased estradiol level at the time of oocyte collection, as also been reported in the bovine dominant preovulatory follicles [19]. This model is physiologically relevant since it is well established that high level of LH (e.g. LH surge) during preovulatory development is associated with marked decrease in follicular and circulatory estradiol levels and that insufficient gonadotropin support results in atresia of the subordinate follicles. In the latter context, withdrawal of gonadotropic support (e.g. anti-eCG antibody treatment) in the present model induced granulosa cell apoptosis and follicular atresia [20-22]. Fertilization and developmental competence of oocytes from anti-eCG treated rats are dependent on the dilution of antibody used (Jiang *et al*., unpublished data).

The objective of the present study is to assess, using an *in vivo *immature rat model, gene expression profile in granulosa cells, which may be linked to the developmental competence of the oocyte. We hypothesized that expression of specific genes in granulosa cells may be correlated with the developmental competence of the oocyte. These findings will facilitate future investigation on the identification of non-invasive biomarkers indicative of oocyte health status which would allow one to select only good-quality oocytes for in vitro fertilization (IVF) and intracytoplasmic sperm injection (ICSI) and to transfer fewer embryos for successful pregnancy.

## Materials and methods

### Materials

All reagents were purchased from Sigma Chemical Company (St. Louis, MO) unless otherwise stated.

### Animal care

Sprague-Dawley rats and New Zealand White Rabbits were purchased from Charles River Canada (Montreal, PQ, Canada). Rats were kept in polycarbonated cages with wood shavings on the floor at 21°C, 50% humidity and a light/dark cycle at 7:00 h/19:00 h. They were given bullet type commercial rat feed and tap water *ad libitum*. The studies were carried out in accordance to the Guide to Care and Use of Experimental Animals of the Canadian Council on Animal Care and approved by the Animal Care Committee of the Ottawa Health Research Institute.

### Production of anti-eCG antiserum

Three male rabbits (2.5 - 3.0 Kg body weight [BW]) were used to produce anti-eCG antisera as described previously [21]. Antibody titres were determined by ELISA. In the bioassay for the antiserum, immature female rats injected with 10 IU eCG were injected 24 h later with highest-titre antiserum or pre-immune serum (100 ul of 1:5 to 1:200 dilution in PBS, i.p.). The ovaries were removed 24 h after treatment and weighed, the ability of various concentrations of the antiserum to prevent eCG-induced ovarian weight gain was assessed. The above dilution (1:5 to 1:200 dilution) of anti-eCG serum significantly decreased ovarian weight in eCG-primed rats.

### Animal treatment and collection of oocytes and mural granulosa cells

Eight immature rats were injected with eCG (10 IU; s.c.; G4877) and 24 h thereafter with either pre-immune serum (control; to stimulate follicle development) or anti-eCG antibody (1:400, to induce follicular atresia). Twenty-four hours later, hCG (10 IU; i.p.; CG-5) was administered. Cumulus-oocyte complexes (COCs) and mural granulosa cells collected by follicle puncture 13 h after hCG were respectively subjected to *in vitro *fertilization or kept at -80°C until the assessment of gene expression, as described hereafter.

### *In vitro *fertilization (IVF) and embryo transfer

To assess the developmental competence of oocytes which were morphologically indistinguishable in both groups, COCs were inseminated in vitro and the fertilized oocytes were transferred into pseudo-pregnant rats as described previously [23]. Briefly, sperm suspensions (1 × 10^6^cells/ml) were pre-incubated in insemination media (400 μl of IVF-30 supplemented with 30 mM NaCl) for 5 to 7 h at 37°C in 5% CO_2 _in air. COCs were then carefully transferred into the suspension drops and incubated for 12 h. The oocytes were transferred into 100 μl of culture medium and freed from surrounding cumulus cells. The denuded oocytes were considered fertilized if they exhibited the presence of pronuclei with sperm tail(s) in the vitellus.

To assess the developmental competence in vivo of embryos fertilized in vitro, nine to ten embryos at the 1-cell stage were transferred to the oviducts of each pseudo-pregnant recipient at Day 1. Vaginal smear of recipients was examined on days 1 and 4 as well as days 12-14 after transfer to confirm successful induction of pseudo-pregnancy and signs of pregnancy, respectively. All recipients were sacrificed by day 24 of pregnancy regardless of delivering offspring, and their uterine horns were examined for implantation sites. The number of young was counted on the day of parturition.

### RNA isolation

Total RNAs from mural granulosa cells collected from ovarian follicles were extracted using RNeasy Mini kit according to manufacturer's instructions and DNA contamination was removed by DNase I digestion (Qiagen Inc., Mississauga, ON, Canada). All total RNA specimens were quantified and checked for quality with a Bioanalyzer 2100 system (Agilent, Palo Alto, CA) before further manipulation.

### Affymetrix GeneChip hybridization and image acquisition

A total of 4 NDC and 4 PDC samples were used, thus requiring a total of 8 GeneChips. The GeneChip hybridization and image acquisition were performed at the Ontario Genome Center. Briefly, two rounds of amplification were carried out to successfully generate sufficient labeled cRNA for microarray analysis from 100 ng of total RNA. For first round synthesis of double-stranded cDNA, total RNA was reverse transcribed using the Two-Cycle cDNA Synthesis kit (Affymetrix) and oligo (dT) 24-T7 (5'-GGCCAGTGAATTGTAATACGACTCACTATAGGGAGGCGG-3') primer followed by amplification with the MEGAscript T7 kit (Ambion, Inc., Austin, TX). After cleanup of the cRNA with a GeneChip Sample Cleanup Module IVT Column (Affymetrix), a second-round double-stranded cDNA was produced using the IVT Labeling kit (Affymetrix). A 15 μg-aliquot of labeled product was fragmented by heat and ion-mediated hydrolysis (94°C, 35 minutes) in 24 μL H_2_O and 6 μL of 5× fragmentation buffer (Affymetrix). The fragmented cRNA was made into hybridization cocktail and was hybridized (16 h, 45°C) to an Affymetrix Rat 230.2 array. Washing and staining of the arrays with phycoerythrin-conjugated streptavidin (Molecular Probes, Eugene, OR) was completed in a Fluidics Station 450 (Affymetrix). The arrays were then scanned using a confocal laser GeneChip Scanner 3000 and GeneChip Operating Software (Affymetrix).

### Microarray data analysis

Gene expression patterns were determined using Affymetrix Genechip Arrays Rat 230.2. Prior to any statistical analysis, raw data were normalized and compared using RMA (robust multichip average) method from the BioConductor package http://www.bioconductor.org, which uses a robust average of log_2_-transformed background-corrected perfect match probe signal intensities combined with a quantile normalization method [24,25]. The quality analysis of the slides was performed by checking the logarithmic scatter plots of probe set intensities in all the non-redundant pairs of replicated samples after the normalization procedure [26]. Normalized data were then filtered in three steps. First, probe sets called 'Absent' (A) over all conditions and replicates across the complete dataset were excluded. Second, a threshold as the 95^th ^percentile of all the absent call signals of the entire dataset was set. All the remaining probe sets whose expression values were consistently below this value were removed in each sample [26]. To extract significant genes between two independent groups, the two-sample t statistic was used for filtered genes. In addition, multiple testing corrections were performed by computing adjusted p values using the Bonferroni and Sidak algorithm which provides experiment-wise (or Family-wise) type I control. Genes with a fold change of 1.2 (increase or decrease) relative to the poor oocyte developmental competence were subsequently used. Hierarchical clustering of samples and gene expression values based on similarities of expression levels was performed using the average linkage method and Euclidean distance measurements as implemented in the TIGR Multiexperiment Viewer (MeV) program [27]. Gene Ontology (GO) analysis was performed with DAVID http://david.abcc.ncifcrf.gov/[28].

Reproducibility between experiments was assessed by calculating the pairwise concordance of presence calls, which was 92.1-97.9%, and by computing the pairwise Adjusted Coefficient of Determination of log-transformed signal intensities (average of 0.952). High correlation of array signals (low intra-experimental group variation) was observed between rat samples within the groups with oocytes showing normal and poor developmental competence (Data not shown).

### Quantitative real-time PCR validation of microarray results

In order to validate the results of microarray, real time RT-PCR analysis was performed on all 8 samples. Briefly, 0.4 μg of total RNAs extracted from mural granulosa cells of each rat ovarian follicles were reverse transcribed in a final volume of 40 μl solution containing First-Strand Buffer, dNTPs, dithiothreitol (DTT), RevertAid Enzyme (Fermentas), and Random Decamer Primers (Ambion, Inc.). Ten representative genes whose expression levels were remarkably changed in microarray (see Table 1) were further validated, they are lysyl oxidase (*Lox*), glycoprotein-4-beta-galactosyltransferase 2 (*Ggbt2*; *UDP-Gal*), nerve growth factor receptor associated protein 1 (*Ngfrap1*), protein disulfide isomerase-associated 5 and 6 (*Pdia5 *and *Pdia6*), myeloid ecotropic viral integration site 1 homolog (*Meis1*), CD83 antigen, lysozyme (*Lyz*), trinucleotide repeat containing 6 (*Tnrc6*), interleukin 13 receptor alpha 1 (*Il13ra1*). Real-time quantitative PCR analyses for those genes were performed using a LightCycler 2.0 System (Roche Diagnostic Corporation) and a QuantiTect SYBR Green PCR kit (Qiagen, Mississauga, ON, Canada). The thermal cycling conditions were comprised of an initial denaturation step at 95°C (15 min) and 40 cycles at 95°C (15 sec), 58°C (20 sec) and 72°C (30 sec). The primer sequence for each gene, their PCR product size, primer location on rat chromosome, and GeneBank access numbers were shown in Table 1. 18S ribosomal RNA was used as control. Target gene expression level was calculated by relative expression ratio (RER) of Normal Developmental Competence (NDC) to Poor Developmental Competence (PDC), all normalized by 18S as described previously [29]. Briefly, the Livak Method (2^-ΔΔCt ^method) was performed by the following formula: 1) Calculate crossing point change of NDC relative to housekeeping gene 18S, ΔCt (NDC) = Ct (target gene, NDC)-Ct (18S, NDC); 2) Calculate crossing point change of PDC relative to housekeeping gene 18S, ΔCt (PDC) = Ct (target gene PDC) - Ct(18S, PDC); 3) Calculate the difference of these changes between NDC and PDC group, ΔΔCt = ΔCt(NDC)-ΔCt(PDC); 4) finally calculate RER = 2^-ΔΔCt^. Fold changes by real-time qPCR in Table 1 were calculated by Mean of RER for NDC over PDC.

**Table 1:** Summary on gene validation by RT-PCR in comparison with gene array results

Gene	Primer sequence	PCR product size (bp)	Location on rat chromosome	GenBank Access #	Fold changes (NDC/PDC) by	
					
					gene array	RT-PCR
Lysyl oxidase	RV:AGTCTCTGACA	129	18q11	NM_017061	2.8	2.86
(*LOX*)	TCCGCCCTA C					
	FW:ACCTGGTACCC					
	GATCCCTA					
Glycoprotein-4-	FW:AGATAAAGATG	186	5q22	NM_053287	-1.7	-1.04
Beta-galactosyltrans	GGCGGCCGTTACT					
ferase 2 (*GGBT2;*	RV:ACATGGTGTCT					
*UDP-Gal*)	CCAGCCTGATTGA					
Nerve growth factor	FW:AATGATGGGTT	175	Xq35	NM_053401	1.6	1.03
receptor associated	GGGTGGAGATGGA					
protein 1 (*Ngfrap1;*	RV:ACCGAAGTCAA					
*Bex3; Nade*)	GGCATAAGGCAGA					
Protein disulfide	FW:ATATGACCGAG	185	11q22	NM_001014125	1.8	1.86
isomerase-	CTGTGACGCTGAA					
associated 5 (*Pdia5*)	RV:ACATCTTTGGC					
	TCCAGGGTCTTCT					
Protein disulfide	FW:ACCTTCTTTCT	182	Chromo-	NM_001004442	1.8	1.04
isomerase-	AGCGGTCAGTGCT		some 6			
associated 6 (*Pdia6*)	RV:AGTGCACTTGC					
	TGCTTTCTTCCAC					
Myeloid ecotropic	FW:TAGCCACCAAT	99	14q22	XM_223643	1.6	1.33
viral integration site 1	ATCATGAGGGCGT					
homolog (*Meis1*)	RV:TGAGTCCCGTA					
	TCTTGTGCCAACT					
CD83 antigen	FW:ATGTGCCTGAA	193	17p12	NM_001108410	1.7	1.6
	TACCACCTGGACA					
	RV:AGCCGCATGAA					
	ACATGAAGCTGAC					
Lysozyme (*Lyz*)	FW:TATGAACGCTG	95	7q22	NM_012771	1.7	1.37
	TGAGTTCGCCAGA					
	RV:TGCTGAGCTAA					
	ACACACCCAGTCT					
Trinucleotide repeat	FW:TGAAGTACCTC	176	1q36	NM_001107549	1.7	1.03
containing 6 (*Tnrc6*)	CACGATTTCGCCA					
	RV:TGCTGTTCTGC					
	ACCTCTCCGTTAT					
Interleukin 13	FW:AAGTGAGAAGC	155	Xq12	NM_145789	1.4	1.16
receptor alpha 1	CTAGCCCTTTGGT					
(*Il13ra1*)	RV:AGTTGGTGTCC					
	GGGCTTGTATTCT					
18S rRNA	FW:CGCGGTTCTAT	219	Chromo-	M11188	Housekeeping	
	TTTGTTGGT		some 3			
	RV:AGTCGGCATCG					
	TTTATGGTC					

### Statistical Analysis

**Figure 1:**
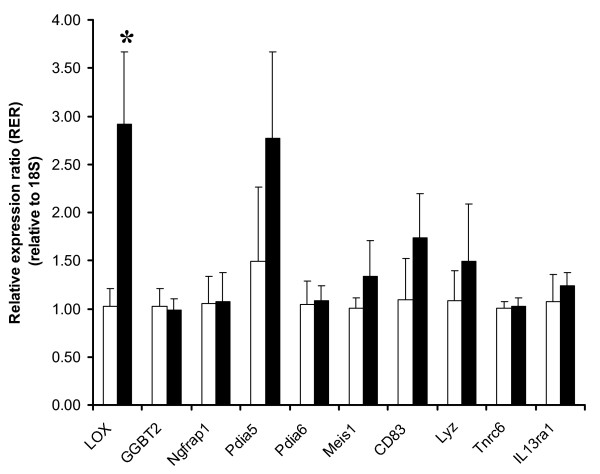
**Validation of differentially expressed genes by real-time qPCR**. Relative quantification of ten representative genes was performed. The method of Livak and Schmittgen (2001) was used to calculate the relative expression ratio (RER) that were normalized to a housekeeping gene 18S. Normal oocyte developmental competence (NDC) (solid bar) were expressed over poor oocyte developmental competence (PDC) (open bar), positive ratio refers to genes up-regulated, negative ratio indicated gene down-regulation, by which real-time qPCR data in the gene regulation trend (up- vs. down-regulation) were consistent with results obtained from microarray, of which the expression level of *Lox *(asterisk) was significantly higher in NDC in comparison to PDC (P < 0.05).

Data in Table 2 and real-time PCR results (Fig. 1) were analyzed by student's t-test tests using Graph Pad Prism 3 software. Differences with P < 0.05 were considered statistically significant.

**Table 2:** In vitro fertilization and embryo transfer of oocytes from immature rats treated with eCG/anti-eCG/hCG

Experimental Group	Rat	Paired Ovarian weight (mg)	No (%). of oocytes/fertilized	No (%). of pups/transferred embryos	No. of pups/implantation Sites
PDC	A	104.0			
	B	109.8			
	C	87.3	18/21(86)	0/10(0)	0/1
	D	131.6	36/37(97)	0/9(0)	0/2
	Mean ± SEM	108.2 ± 7.9	(93.5 ± 2.7)	(0)	0/4 ± 2

NDC	E	98.7	23/23(100)	4/10(40)	4/6
	F	104.9	26/28(93)	5/10(50)	5/8
	G	79.9	12/12(100)	3/10(30)	3/4
	H	89.5	18/20(90)	3/10(30)	3/6
	Mean ± SEM	93.3 ± 4.7	(95.8 ± 2.2)	(37.5 ± 4.2)	4 ± 1/6 ± 1

PDC: Oocytes with poor developmental competence; NDC: Oocytes with normal developmental competence.

## Results

### Production of oocytes with poor and normal developmental competence

Treatment of eCG-primed rats with low dose of anti-eCG antiserum (1:400 dilution) failed to significantly decrease paired ovarian weight (108.2 ± 7.9 mg versus 93.3 ± 4.7 mg; P > 0.05) and fertilization rates (93.5 ± 2.7% versus 95.8 ± 2.2%, P > 0.05) when compared with those in eCG plus pre-immune serum-treated group (Table 2). However, anti-eCG antiserum injection resulted in the production of oocytes with poor developmental competence. No embryos in this group could develop to term after embryo transfer. In contrast, as high as 30%-50% of oocytes from eCG-primed rats developed to offspring (P < 0.05). No significant differences in the number of implantation sites were observed between two groups (Table 2).

### Microarray identification of differentially expressed genes

**Figure 2:**
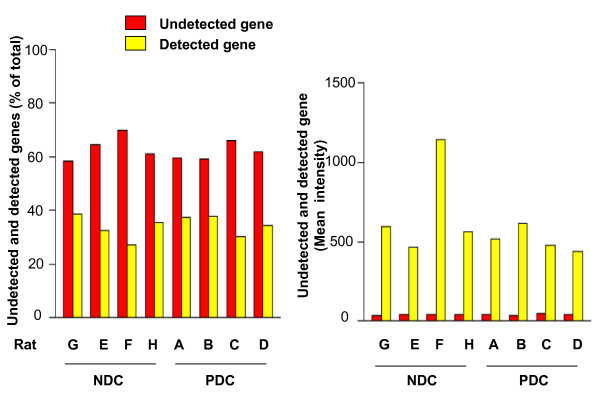
**Percentages (left panel) and mean gene expression intensities (right panel) of detected and undetected genes in 8 gene arrays**. The number of undetected genes was higher than that of detected genes in all arrays (left panel). However, the mean gene expression intensities of detected genes were much higher than those of undetected genes in all arrays (right panel).

The global gene expression profiles in rat granulosa cell samples representing oocytes of poor and normal developmental competence were identified with microarray technique. Results in Fig. 2 (left panel) show that among the approximately 30,000 genes queried on Rat 230.2 array, there were more undetected genes than detected genes observed in all arrays. Mean expression intensities of detected genes were higher than those of undetected genes (Fig. 2, right panel). A log_2 _signal intensity threshold of 98.3 was determined and only those genes with signal intensity smaller than 98.3 were filtered. 8985 genes were left for further analysis.

**Figure 3:**
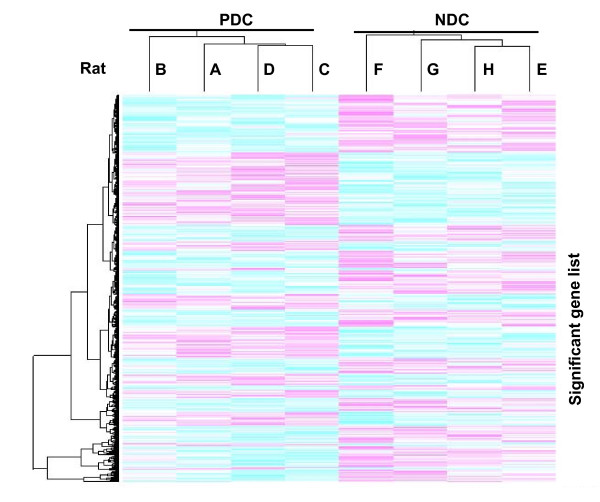
**Unsupervised hierarchical clustering analysis of 701 differentially expressed probe sets in all arrays**. To identify the relationships between samples, a 1 - correlation metric with centroid linkage was applied to those probe sets. A dendrogram containing two distinct arms was identified. All four samples from poor oocyte developmental competence (PDC) group had similar gene expression patterns and were included in the same PDC cluster. On the other hand, all other four samples from normal oocyte developmental competence (NDC) group had similar gene expression patterns and were included in the same NDC cluster. The gene expression patterns were very different between PDC and NDC clusters.

Of a total of about 30,000 probe sets, we observed that the expression of 701 genes (Table 3) were significantly different (P < 0.001) between oocytes with poor developmental competence compared to normal one, 43 of which were altered > 1.2-fold, and 13 of which > 1.5-fold. Both up- or down-requlated genes are shown in Table 3. A Euclidean clustering of these differential genes is shown in Fig. 3. All four samples from poor oocyte developmental competence (PDC) group had similar gene expression patterns and were included in the same PDC cluster. On the other hand, all other four samples from normal oocyte developmental competence (NDC) group had similar gene expression patterns and were included in the same NDC cluster. The gene expression patterns were very different between PDC and NDC clusters.

**Table 3:** Expression and their biological functions of genes in mural granulosa cells of follicles containing oocyte with normal developmental competence compared to those with poor developmental competence, as determined by Gene Ontology Analysis

Probe position at array	Fold changes*	Gene	Biological functions
**Transcription regulation genes**			

1367847	1.8	Nuclear protein 1	Unknown
1384308	1.6	Meis1 (myeloid ecotropic viral integration site 1 homolog)	Regulation of transcription, DNA dependent
1371947	1.6	Necdin	Unknown
1371822	1.5	RNA polymerase III (DNA directed) polypeptide D	Regulation of progression through cell cycle
1375414	1.5	TAF9 RNA polymerase II [TATA box binding protein (TBP)-associated factor]	Negative regulation of transcription from RNA polymerase II promoter
1390116	1.4	Transcribed locus: similar to polymerase I-transcript release factor (PTRF)	Unknown
1374780	1.3	Transcribed locus	Unknown
1372093	1.3	Max interacting protein 1	Unknown
1373978	1.3	Nuclear cap binding protein subunit 1 (80 kDa)	RNA splicing and Mrna cleavage
1385486	1.3	Transcribed locus	Unknown
1380827	1.3	Similar to C1orf25	tRNA processing
1370826	1.3	Nucleosome assembly protein 1-like 1	DNA replication, nucleosome assembly and positive regulation of cell proliferation
1376597	1.3	Ninc finger, CCHC domain containing 10	Unknown
1388067a	-1.3	Glucocorticoid modulatory element binding protein 2	Regulation of transcription, transcription from RNA polymerase II promoter

**Post-translation regulation genes**			

1368171	2.8	Lysyl oxidase	Protein modification, copper ion binding oxidoreductase activity, cancer metastasis, granulosa cell differentiation
1374828	1.8	Protein disulfide isomerase-associated 5	Electron transport, protein folding and response to stress
1370859	1.5	Protein disulfide isomerase associated 6	Electron transport, protein folding and electron transport
1398895	1.4	Golgi autoantigen, golgin subfamily a,7	Protein amino acid palmitoylation
1392149	1.3	Transcribed locus	Unknown
1368653a	1.3	Parkinson disease (autosomal recessive, early onset) 7	Protein folding, cell proliferation and adult locomotory behavior
1387258a	1.3	Protein-L-isoaspartate (D-aspartate) O-methyltransferase 1	Protein methylation, S-adenosylhomocysteine metabolism and protein modification
1386164	1.3	Cell division cycle 2-like 5 (cholinesterase-related cell division controller)	Protein phosphorylation, regulation of mitosis and positive regulation of cell proliferation
1398343	1.2	DNAJ (Hsp40) homolog, subfamily A, member 4	Protein folding
1383475	-1.3	Protein phosphatase 1A, magnesium dependent, alpha isoform	Protein dephosphorylation, positive regulation of IkB kinase/NFkB cascade

**Microtubule cytoskeleton regulation genes**			

1370154	1.7	Lysozyme	Antimicrobial activity in human follicular fluid, ovulation
1390529	1.7	CD83 antigen	Defense response, humoral immune response and signal transduction
1375664	1.7	Trinucleotide repeat containing 6	Microtubule-based movement
1369948	1.6	Nerve growth factor receptor associated protein 1	Induction of apoptosis, increase in PCO ovaries
1374321	1.4	Similar to RIKEN cDNA 2700081O15	Unknown
1388711	1.4	Interleukin 13 receptor, alpha 1	Cell surface receptor linked signal transduction
1372330	1.4	Goliath	Apoptosis and proteolysis
1372682	1.3	Similar to RIKEN cDNA 2810432L12	Unknown
1372093	1.3	Max interacting protein 1	Unknown
1386952a	1.3	Dynein, cytoplasmic, intermediate chain 2	Microtubule-based movement
1380577	1.3	ATP-binding cassette, sub-family G (WHITE), member 2	Drug transport
1369970	1.3	Vesicle-associated membrane protein 8	Protein complex assembly and vesicle-mediated transport
1367716	1.2	T-cell immunomodulatory protein	Unknown
1373090	1.2	Signal sequence receptor, alpha	Cotranslational protein targeting to membrane, positive regulation of cell proliferation
1376874a	1.2	Adaptor-related protein complex AP-4, beta 1	Intracellular protein transport, vesicle-mediated transport
1383206	1.2	Component of oligomeric golgi complex 3	Intracellular protein transport
1369549	-1.3	Killer cell lectin-like receptor subfamily K, member 1	Unknown
1371073	-1.7	UDP-Gal: betaGlcNAc beta 1,4-galactosyltransferase, ploypeptide 1	Promote apoptosis, N-acetyllactosaminesynthase activity, beta-N-acetylgluco-saminylglycopeptide beta-1,4-galactosyltransferase activity, carbohydrate metabolism, development of secondary sexual characteristics, extracellular matrix organization and biogenesis, galactose metabolism, integral to membrane, lactose synthase activity, oligosaccharide biosynthesis, transferase activity,

*Fold changes represent difference of gene expression in granulosa cells from follicles containing oocytes with normal developmental competence compared with that with poor developmental competence. "-": down-regulation; others: up-regulation

### Gene ontology analysis

Gene ontology analysis showed that up-regulated genes in oocytes with normal developmental competence were linked to transcription regulation, protein phosphorylation and signal transduction, microtubule cytoskeleton organization and movement (Table 3). The genes participating in transcriptional regulation included nucleosome assembly protein 1-like 1, *Necdin, Meis 1 *and TAF9 RNA polymerase II and a transcribed locus homologous to polymerase I-transcript release factor (*PTRF*), while those involved in the control of protein phosphorylation and signal transduction were *Lox, Pdia5 *and *Pdia6*, golgi autoantigen and cell division cycle 2-like 5. The genes having a role in microtubule cytoskeleton organization and movement include CD83 antigen, *Tnrc6, Goliath*, vesicle-associated membrane protein 8 (Table 3).

Twelve genes were up-regulated, and one gene down-regulated, more than 1.5 folds in NDC group than those in PDC group. Gene ontology classification showed that the up-regulated genes included *Lox *and *Ngfrap1*. *Lox *is important in the regulation of copper ion binding [30]. *Ngfrap1 *plays an important role in apoptosis induction [31]. The down-regulated gene is *Ggbt2 *known to be involved in the regulation of extracellular matrix organization and biogenesis [32].

### Identification of signaling pathways contributing to the normal oocyte developmental competence

To determine the signaling pathways of up-regulated genes associated with normal oocyte developmental competence, all genes with more than 1.2-fold change were subjected to the pathway analysis by Pathwayexplorer https://pathwayexplorer.genome.tugraz.at/. Although no directly related pathways were found, a potential signaling pathway of the highest-regulated gene, *Lox*, could be envisaged since oocyte-derived factors such as GDF-9 increases gene expression of *Lox *which induces differentiation of mural granulosa cells [33].

### Quantitative real-time PCR validation of microarray data

Ten representative genes, the expression levels of which were remarkably changed in microarray (Table 3), were selected for further validation by RT-PCR analyses. Of ten genes selected, *Lox, Pdia5*, and CD83 antigen mRNA abundance of mural granulosa cells in normal oocyte developmental competence group were higher (fold changes > 1.6) than that in poor oocyte developmental competence group, consistently in both gene microarray and quantitative RT-PCR analyses. The fold change from microarray and that from RT-PCR exhibit excellent concordance, with Pearson correlation equal to 0.94 (p < 0.0001). However, only *Lox *was statistically significantly different between the two groups (fold changes > 2.8, P < 0.05, Fig. 1 and Table 1). Our data suggested that the profile of *Lox *gene in mural granulosa cells could be a likely candidate for a potential biomarker for follicular maturity and oocyte quality.

## Discussion

In the present study, using whole genome gene expression profiling of mural granulosa cells, we have demonstrated that mural granulosa cells isolated from follicles containing oocytes with normal developmental competence are distinct from those with oocytes exhibiting poor developmental competence. The dissimilarity between these two groups was clearly shown through unsupervised hierarchical clustering of these samples and was substantiated using binary tree prediction as well as expression data from independent arrays. The identification of two unique branches containing normal and poor oocyte developmental competence is consistent with the distinct developmental outcome after embryo transfer. Meanwhile, our comparison of gene expression profiles between different samples within the same group showed that there was a high "within-group" similarity, demonstrating the quality of our gene expression experiment. Differentially expressed genes in these two groups might be further tested as potential biomarkers of oocyte quality, in particular the highest changed gene encoding lysyl oxidase that plays an important role in the regulation of differentiation of mural granulosa cells.

The assessment of differential gene expression between two groups, in conjunction with gene ontogeny analysis, showed that differences in genes were associated with regulation of transcription and DNA replication and cell cycle progression, protein folding, phosphorylation and signaling pathways, microtubule cytoskeleton organization and movement, and receptor signaling and apoptosis. Of principal importance was the gene "*Lox*" which, with the largest difference in expression, has been shown to be involved in the regulation of mural granulosa cell differentiation. *Lox *was expressed 2.8-fold higher in mural granulosa cells in follicles producing normal oocyte than poor oocyte developmental competence. This enzyme oxidizes peptidyl lysine to peptidyl aldehyde residues within collagen and elastin, initiating formation of the covalent cross-linkages that insolubilize these extracellular proteins [34]. This enzyme is also present and active within rat vascular smooth muscular cell nuclei, exhibits its catalytic activity on histone H1 [35,36], suggesting that it may regulate chromatin remodeling involved in the regulation of transcription [37]. It has been shown that *Lox *is expressed in cultured bovine granulosa cells and involved in the maintenance of cell differentiation [30]. The activity of this enzyme is increased in rabbit ovarian follicles after hCG-induced ovulation and its mRNA expression is up-regulated at the time of ovulation in perch ovary [38,39]. However, rat granulosa cell *Lox *transcripts were significantly suppressed 48 h after eCG injection compared with untreated controls and were further reduced during hCG-induced luteinization [38]. Furthermore, FSH dose-dependently inhibited *Lox *mRNA and enzyme activity in cultured rat granulosa cells [33].

In the present study, *Lox *mRNA abundance was 2.8-fold higher in mural granulosa cells isolated from follicles containing oocytes which exhibit normal developmental competence when compared with poor ones. This result was validated by real-time PCR. It has been demonstrated that TGFβ1 and GDF9 increase *Lox *mRNA expression in human lung fibroblasts [40] and rat granulosa cells [33], respectively. Since the actions of TGFβ superfamily members are mediated via the Smad2/Smad3 pathways [33], these findings raise the interesting possibility that the GDF9-induced preantral follicular growth in vitro [12] involves increased mural granulosa cell *Lox *mRNA expression. Whether this indeed is the case awaits further investigation.

In addition to *Lox, Pdia5 *is also up-regulated at less extent in the normal oocyte developmental competence group. Although *Pdia5 *plays an important role in the regulation of electron transport, protein folding and stress response [41], posttranslational protein modification and is essential for normal cell function [42], the differences between the two experimental groups are not statistically significant as determined by real-time PCR. The physiological significance of this observation remains unclear.

## Conclusions

The present studies demonstrate a close association between the expression of *Lox *in mural granulosa cells and the developmental competence of oocytes. These findings suggest that the most diffentially expressed gene, lysyl oxidase, may be a potential biomarker for oocyte health in assisted reproduction. Further studies are required to confirm this notion.

## Competing interests

The authors declare that they have no competing interests.

## Authors' contributions

JYJ designed the experiment, conducted animal studies (including development and injection of eCG-antibody, IVF/embryo transfer and collection of cells), extracted RNA and prepared manuscript. HX analyzed gene array data and was assisted by XX. MC performed real-time RT-PCR. MAS assisted in experimental design. BKT involved in designing this study and developing the manuscript. All authors have read and approved the final manuscript.

## Funding

This work was supported in part by a grant from the Canadian Institutes of Health Research (MOP-10369) and by the World Class University (WCU) program (R31-10056) through the National Research Foundation of Korea funded by the Ministry of Education, Science and Technology. In addition, the studies described were part of the Program on Oocyte Health http://www.ohri.ca/oocyte funded under the Healthy Gametes and Great Embryos Strategic Initiative of the Canadian Institutes of Health Research (CIHR) Institute of Human Development, Child and Youth Health (IHDCYH), grant number HGG62293. J.Y.J. and M.C. are recipients of CIHR-STIRRHS Postdoctoral Fellowships.
